# HLA-B*27 is associated with CNO in a European cohort

**DOI:** 10.1186/s12969-023-00826-7

**Published:** 2023-06-05

**Authors:** Daire O’Leary, Dalila Ali Al Julandani, Muhammad Zia, Jens Klotsche, Kirsten Minden, Marion Roderick, Athimalaipet V. Ramanan, Orla G. Killeen, Anthony G. Wilson

**Affiliations:** 1 Centre for Arthritis Research, Conway Institute, School of Medicine, University College Dublin, Belfield, Dublin 4, Ireland; 2 National Centre for Paediatric Rheumatology, Children’s Health Ireland, Dublin, Ireland; 3 Department of Paediatric Rheumatology, Bristol Royal Hospital for Children, Bristol, UK; 4 Deutsches Rheuma-Forschungszentrum Berlin, Leibniz Institute, Berlin, Germany; 5 Department of Pediatric Respiratory Medicine, Immunology and Critical Care Medicine, Charité Universitätsmedizin Berlin, corporate member of Freie Universität Berlin and Humboldt-Universität Zu Berlin, Berlin, Germany; 6 Department of Paediatric Immunology and Infectious Diseases, Bristol Royal Hospital for Children, Bristol, UK; 7 Population Health Sciences, University of Bristol, Bristol, UK; 8 Translational Health Sciences, University of Bristol, Bristol, UK

**Keywords:** Paediatric rheumatology, Chronic nonbacterial osteomyelitis, CNO, CRMO, HLA

## Abstract

**Objectives:**

To determine the influence of HLA-B27 positivity on risk of developing chronic nonbacterial osteomyelitis (CNO).

**Methods:**

HLA-B*27 genotype was assessed in 3 European CNO populations and compared with local control populations (572 cases, 33,256 controls). Regional or whole-body MRI was performed at diagnosis and follow-up in all cases which reduces the risk of disease misclassification. Genotyping was performed using either next generation DNA sequencing or PCR based molecular typing. Statistical analysis used Fisher’s exact test with Bonferroni correction and a fixed effects model for meta-analysis of odds ratios.

**Results:**

HLA-B*27 frequency was higher in all 3 populations compared with local controls (combined odds ratio (OR) = 2.2, *p*-value = 3 × 10^–11^). This association was much stronger in male compared with female cases (OR = 1.99, corrected *p*-value = 0.015). However, the HLA-B*27 status was not statistically significantly associated with co-occurrence of psoriasis, arthritis or inflammatory bowel disease.

**Conclusion:**

Carriage of HLA-B*27 is associated with greater risk of developing CNO, particularly in male cases.

## Introduction

Chronic nonbacterial osteomyelitis (CNO) is a rare autoinflammatory bone disease (OMIM 259,680), predominantly affecting children and adolescents with an estimated prevalence of 1 per 10^5^–10^6^ [1]. It is characterised by relapsing episodes of localised bone inflammation, most commonly affecting long bone metaphyses, clavicles, vertebrae and pelvis [1]. Long-term sequelae can include psychosocial complications, scoliosis and leg-length discrepancy [1]. It is associated with other inflammatory conditions, in particular inflammatory arthritis, psoriasis and inflammatory bowel disease, although the reported co-occurrence of these conditions varies widely [1–3].

HLA-B*27 testing is recommended in the Childhood Arthritis and Rheumatology Research Alliance (CARRA) consensus treatment and monitoring recommendations due to the possible association between CNO and HLA-B*27 associated inflammatory disease [4].

There is controversy regarding the role of HLA-B*27 in CNO. While an association was proposed almost 20 years ago, many subsequent studies fail to replicate this. However, of the 17 studies both supporting and refuting this association, 14 involved HLA-B*27 testing in 43 or fewer participants (range 6 – 43) [3, 5–10]. Three studies involved HLA-B*27 typing in 72, 86 and 163 participants; none showed an association with CNO [1, 11, 12]. In addition, the control population data and ethnicity of participants is not reported in all studies; ethnicity may be of particular relevance when looking at small studies. Therefore, the relationship between HLA-B*27 and CNO remains unclear.

In this study we examined HLA-B*27 status in three European CNO populations compared with local control populations. We also assessed whether HLA-B*27 was associated with major demographic or clinical variables. Our data reveal this variant to be a risk allele for CNO, particularly in male cases.

## Patients and methods

### Study population

Three European cohorts of children with CNO were included in the study. The Irish cohort included children attending the National Centre for Paediatric Rheumatology in Children’s Health Ireland between September 2018 and December 2021. The Bristol cohort included children who attended Bristol Children’s Hospital between 2010 and 2021. All patients in the Irish and Bristol cohorts met the Bristol criteria for CNO. All MRI scans were assessed and reported on by a radiologist as part of the diagnostic process, independent of this study. The German National Paediatric Rheumatologic Database (NPRD) includes patients from over 60 centres in Germany and Austria. Since 2009, it has gathered clinical and sociodemographic information on CNO from clinicians and patients using standardised questionnaires. A confirmed diagnosis of CNO by an expert was required for inclusion in the NPRD. Confirmation of diagnosis for the NPRD was based on clinically symptomatic inflammatory lesions with the exclusion of other relevant diagnoses as described in the previously published one-year outcome data, with diagnosis confirmed by the same authors (CR, KM, HG) for all cases [13]. The same methodology was applied to any additional cases recruited from 2018–2020 which were included in this study but not described in that paper [13]. CNO data recorded in the NPRD between 2016 and 2020 were included in this study with the most recent entry extracted for each patient.

### Ethics approval

The study received ethics approval from Children's Health Ireland at Crumlin (GEN/572/17) and Charite Medical University of Berlin (NPRD). Data collection for this study received an ethics exemption from Bristol University Hospital (UK). Informed consent/assent was obtained locally at CHI and NPRD sites.

### Data collection

Detailed demographic, clinical, laboratory and radiological data were recorded on all patients. MRI diagnosis of lesions was based on the presence of bone marrow hyperintensity, bony expansion, soft tissue hyperintensity and complications such as vertebral compression or pathological fracture and the exclusion of alternative diagnoses such as enthesitis-related arthritis (ERA). Whole-body MRI data was included for all participants in the Irish and Bristol cohorts but was not available from the NPRD cohort. Ethnicity was recorded in the Irish and Bristol cohorts but not in the NPRD cohort. Ethnicity was self-reported in response to an open question.

### HLA typing in the Bristol and NPRD cohorts

Molecular HLA-B*27 typing using polymerase chain reaction (PCR) was performed on patients attending tertiary paediatric rheumatology services at centres participating in the NPRD at the treating clinician’s discretion. All patients attending Bristol Children’s Hospital (UK) are being tested using PCR typing and HLA-B*27 typing is being requested on all patients as part of routine phlebotomy regardless of the site of CNO lesion or co-morbidities. Patients who had HLA-B*27 testing completed prior to 31.3.2022 were included in this study.

### HLA prediction in the Irish cohort

Whole exome sequencing was performed on genomic DNA from whole blood as part of an ongoing study. Samples were sent to either University of Leeds Next Generation Sequencing laboratory or Novogene UK for whole exome sequencing using Illumina HiSeq 3000 and NovaSeq 6000, respectively, with 150 bp paired-end reads. Reads were aligned to the hg19 reference genome using BWA software, duplicates removed using Picard tools and GATK software used to realign indels and call variants. HLA alleles were predicted from whole exome sequencing results using Optitype software [14] through the Nextflow nf-core/hlatyping pipeline (version 1.1.5). Molecular PCR-based HLA typing in the same cohort was performed at the treating clinician’s discretion.

### Population control data

HLA imputed data was obtained from the UK Biobank (project number 80917) [15]. HLA imputation from single nucleotide polymorphism (SNP) data was performed in the UK Biobank as previously described using the HLA*IMP:02 algorithm [15, 16]. Self-reported ethnicity was used to match the Irish and Bristol cohorts with an appropriate UK Biobank control population; all cases and controls for the Irish cohort were ethnically Irish while the proportion of different ethnicities in the Bristol cohort were matched to the UK Biobank data. Data from the German Bone Marrow Registry publicly available on the Allele Frequency Net Database (AFND), accessed at allelefrequencies.net [17], was used as control data for the NPRD cohort. HLA typing on this cohort used reverse sequence specific oligonucleotide probe (SSOP) and included participants are those who self-report as German living in Germany.

### Statistical analysis

Statistical analysis was performed in Rstudio (version 1.1.456). Fisher’s exact test was performed to determine odds ratios (OR) and 95% confidence interval (95% CI) using R package *stats* (version 4.1.0). Bonferroni correction was used to correct for multiple testing. Heterogeneity between study populations was tested using Cochrane’s Q test and I^2^ statistic. Significant heterogeneity was defined as a Q test *p*-value < 0.01 and/or an I^2^ > 50%; a fixed effect model is appropriate for meta-analysis if there is a lack of significant heterogeneity. A fixed effects model was used for the meta-analysis of odds ratios using R package *meta* (version 5.2.0).

### Patient and public involvement

Patients were not involved in the study design. A patient research partner was involved in developing plain English information regarding the Irish arm of the study. After publication, results will be disseminated through a plain English newsletter, co-edited by a patient research partner.

## Results

Detailed phenotypic data from the NPRD [13] and Irish cohorts [18] were previously published; all cohorts are similar to previously published CNO cohorts in terms of median age of onset, female predominance, multifocal disease and co-morbidities. Phenotypic data for the cohorts is summarised in Table 1.

**Table 1:** Characteristics of three European cohorts

Cohort *n* = number tested for HLA-B*27	HLA-B*27 n (%)	Median age at onset (years)	Median follow up (years)	Multifocal n (%)	Male n (%)	Psoriasis n (%)	Arthritis n (%)	IBD n (%)
Germany *n* = 476	79 (16.5)	10.34	2.49	291 (61)	173 (36.4)	32 (6.7)	88 (18.5)	17 (3.6)
Ireland *n* = 50	10 (20)	9.23	2.75	46 (92)	14 (28)	9 (18)	9 (18)	1 (2)
Bristol *n* = 46	6 (13)	10.0	2.35	39 (87)	13 (28.3)	3 (6.5)	8 (17.4)	0 (0)

All patients in the Irish and Bristol cohorts underwent whole-body MRI. CNO lesions were most frequently identified in the long bones of the lower limbs in all cohorts; the distribution of lesions in the Irish and Bristol cohorts is shown in Table 2. In the Irish and Bristol cohorts, 0/9 and 6/8 of those with inflammatory arthritis had axial involvement, respectively. 2/8 in the Bristol cohort met the criteria for enthesitis-related arthritis (ERA) during follow-up; these patients met criteria for CNO clinically and radiologically but subsequently developed sacroiliitis clinically and on imaging. None of the Irish cohort met the criteria for ERA. In those with axial arthritis/ERA in these cohorts, all had MRI signs consistent with CNO at sites distant from entheseal points, in addition to lesions at sites not classically associated with ERA such as clavicular or mandibular lesions. Those with peripheral arthritis had clinical or radiologic signs of synovitis distant from any documented bony lesion.

**Table 2:** Distribution of CNO bony lesions in each cohort

Site	Cohort	Number of participants (%)
Lower limb long bones	Ireland	48 (96)
	Bristol	38 (84.4)
Upper limb long bones	Ireland	15 (30)
	Bristol	17 (37.8)
Axial skeleton	Ireland	17 (34)
	Bristol	17 (37.8)
Foot	Ireland	18 (36)
	Bristol	18 (40)
Vertebra	Ireland	8 (16)
	Bristol	11 (24.4)

**Fig. 1:**
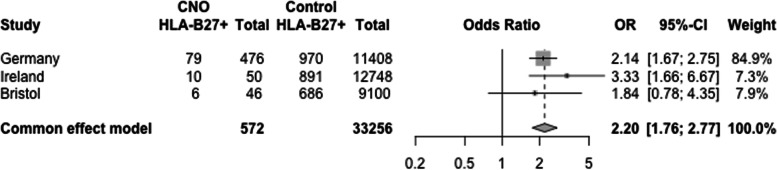
HLA-B*27 is associated with CNO. Forest plot of pooled odds ratios using a fixed effects model (*p* = 3.07 × 10^–11^)

In the Irish cohort, HLA class I prediction was successful from all 50 WES samples. Molecular HLA-B*27 typing was performed in 45/50 of this cohort and agreed with OptiType prediction in all cases. Meta-analysis of the pooled odds ratios from the three European cohorts is shown in Fig. 1.

HLA-B*27 was not associated with an increased risk of psoriasis or inflammatory bowel disease associated with CNO after a median of 2.5 years of follow-up. There was a non-significant trend toward association with an increased risk of inflammatory arthritis. However, there was a statistically significant association between HLA-B*27 and CNO in male compared with female patients (Table 3).

**Table 3:** HLA-B*27 is a risk allele for CNO in males

HLA-B*27 positivity	OR (95% CI)	*p*-value	Corrected *p*-value
Male sex	1.99 (1.24–3.2)	0.0029	0.015
Multifocal disease	0.9 (0.46–1.76)	0.76	1
Arthritis	1.98 (1.13–3.42)	0.012	0.059
Psoriasis	0.82 (0.27–2.04)	0.83	1
Inflammatory bowel disease	0.62 (0.07–2.7)	0.75	1

The previously reported association between HLA-B*27 and CNO affecting bones of the foot [13] was not replicated in the Irish and Bristol cohorts, although there was a non-significant trend toward association in the Irish cohort.

## Discussion

This is the first multicentre study to describe a possible genetic risk factor in sporadic CNO. HLA-B*27 is known to be associated with an increased risk of inflammatory diseases including uveitis and spondyloarthritis with the HLA-B*27 positive subtype of these diseases more frequently associated with male sex. HLA-B*27 is associated with a more chronic and extensive disease course in male children and adolescents with enthesitis-related arthritis (ERA) and with a better response to anti-TNF treatment in ankylosing spondylitis [19, 20]. In acute anterior uveitis, differing cellular infiltrates were recently identified in the aqueous humor of HLA-B*27-positive compared to HLA-B*27-negative patients [21].

The relationship between CNO and HLA-B*27-associated inflammatory disease, particularly enthesitis-related arthritis (ERA) or ankylosing spondylitis, is unclear. An association between CNO and inflammatory arthritis affecting a joint distant from the bony lesion has been reported in several studies [3, 12, 18]. However, most studies do not state whether the arthritis is axial or peripheral. Furthermore, in CNO-associated arthritis, particularly those meeting diagnostic criteria for ERA, there is a risk that MRI signs of enthesitis could mimic CNO lesions leading to misclassification. Individual-level MRI results and detailed phenotypic data were reviewed for all patients in the Irish and Bristol cohorts to mitigate the risk of bias being introduced in this way. Although detailed MRI data was not available from the NPRD cohort for this study, data from participants which included the subset described here were reviewed by three authors of a previous study in order to confirm the diagnosis of CNO [13]. Many rheumatologists believe that CNO and the related condition Synovitis Acne Pustulosis Hyperostosis Osteitis (SAPHO) syndrome belong to the same spectrum of disease as ERA/ankylosing spondylitis [22] with many current CNO treatments extrapolated from ERA treatment pathways [23]. The trend toward association between HLA-B*27 positive CNO and inflammatory arthritis seen in this study requires longer term follow-up to determine disease evolution over time. This follow-up is ongoing and may add clarity to the relationship between CNO and ERA/ankylosing spondylitis.

Recent data from the 1-year follow up of the German NPRD showed a statistically significant association between HLA-B*27 and both involvement of the bones of the foot and progression to second-line treatment with a disease modifying antirheumatic drug (DMARD) during the first 12 months of follow-up [13]. These findings were not replicated in the other two cohorts in this study. It is important to note, however, that the absence of consensus treatment plans prior to 2018 [4], with resultant differences in local prescribing practices, makes it difficult to interpret the rate of progression to second-line treatment between different centres.

One limitation of this study is the relatively short duration of follow-up. This is of particular importance in terms of establishing whether there are differences in disease course dependent on HLA-B*27 status. In addition, it is well established that the rate of HLA-B*27 varies significantly between ethnic groups. Therefore, the use of a control population based on ethnicity rather than geographic location for the NPRD cohort would be preferable. Patients from the NPRD and Bristol cohorts were included on the basis of completed HLA-B*27 typing. While no clear phenotypic differences were seen between this subset and the overall CNO population in these centres, this is another limitation of the study. Testing in the Bristol cohort was performed irrespective of the sites involved in CNO or presence of co-morbidities. However, testing in the NPRD cohort was determined by the treating clinician and, therefore, may have resulted in selection bias. Within the Irish cohort, the 50 who responded to the invitation to participate did not differ in terms of demographics or phenotype from those who did not respond.

## Conclusion

Further research is required to establish whether the HLA-B*27 positive subtype of CNO is phenotypically different from HLA-B*27 negative disease. Based on other HLA-B*27-associated diseases, there may be differences in treatment response dependent on HLA-B*27 status. It would be of interest to establish whether the inflammatory infiltrate differs from HLA-B*27 negative CNO. A comparative study of bone biopsy samples may help to establish whether HLA-B*27 positive CNO is a pathologically distinct disease, as is the case in acute anterior uveitis [21]. Overall, this study supports consistent inclusion of HLA-B*27 typing in patients with CNO in order to better understand its role in disease pathogenesis.

## Data Availability

Data are available upon reasonable request. Deidentified datasets are available from Dr O’Leary (ORCID 0000–0001-9201–8221). Control population data from the UK Biobank is available by applying to directly to the UK Biobank.

## Abbreviations

CNO: Chronic nonbacterial osteomyelitis
NPRD: National Paediatric Rheumatologic Database
AFND: Allele Frequency Net Database
ERA: Enthesitis related arthritis
DMARD: Disease modifying antirheumatic drug
CARRA: Childhood Arthritis and Rheumatology Research Alliance
OR: Odds ratio
CI: Confidence interval
SAPHO: Synovitis Acne Pustulosis Hyperostosis Osteitis
